# Selections

**Published:** 1895-02

**Authors:** 


### Selections.

### A NEW METHOD OF REDUCING DISLOCATION OF THE JAW.

Dr. Roth seats the patient in an ordinary chair, and stands before him with one foot placed slightly to the right side and the other just in front of the patient and in the middle line. He then flexes himself at the hips and causes the patient to lean forward and to place his forehead at the middle of the operator's sternum, —but this position varies with the size of the patient's head. The operator now flexes his neck so that his chin grips the patient's head about the upper part of the occipital bone, thus acquiring a firm hold with the head well under control between his chin and chest. Now the thumbs, protected in the usual manner, are placed in the patient's mouth, and the fingers of both hands grasp the lower jaw.—*The Medical Age.*

